# Legal Decision Biases in GPT: A Comparison with Human Judgment

**DOI:** 10.3390/bs16030437

**Published:** 2026-03-17

**Authors:** Toscane F. Bessis, Andy J. Wills, Bartosz W. Wojciechowski, Lee C. White, Emmanuel M. Pothos

**Affiliations:** 1 Department of Psychology, City St George’s, University of London, London EC1V 0HB, UK; emmanuel.pothos.1@citystgeorges.ac.uk; 2 School of Psychology, University of Plymouth, Plymouth PL4 8AA, UK; andy.wills@plymouth.ac.uk; 3 Institute of Applied Psychology, Jagiellonian University, 30-348 Kraków, Poland; b.wojciechowski@uj.edu.pl; 4 Organisational Behaviour, INSEAD, Singapore 138676, Singapore; lee.white2@insead.edu

**Keywords:** large language models, GPT, legal decision-making, cognitive biases, order effects, bias mitigation, prompt engineering

## Abstract

Legal decision-making is expected to meet high standards of consistency and rationality, yet human judgments in this domain are known to be influenced by procedural factors such as evidence order and intermediate evaluations. Recent work has shown that even legal professionals, including judges, are susceptible to such biases when assessing criminal cases. This raises a critical question: do large language models, which are increasingly proposed as decision-support tools in legal contexts, exhibit similar procedural biases—and if so, can these biases be mitigated? To address this question, we tested GPT-4o and GPT-5.2 using a controlled legal judgment task adapted from prior human research. The task involved simplified criminal cases in which we systematically manipulated (i) the order of incriminating and exonerating evidence and (ii) whether an intermediate guilt judgment was required before a final decision. Model responses were directly compared to human judgments from the original study. We additionally examined whether prompt engineering strategies, based on current best-practice recommendations, could reduce observed biases. GPT-4o exhibited robust order effects and a form of evaluation bias, although the latter differed in structure from the human pattern. GPT-5.2 showed similar but attenuated effects. Across both models, prompt engineering had limited and inconsistent impact, failing to reliably eliminate procedural sensitivity. These findings suggest that even advanced large language models remain vulnerable to normatively irrelevant procedural influences. More broadly, they advise caution in treating large language models as inherently rational or bias-resistant decision-support systems in high-stakes professional domains such as law.

## 1. Introduction

Large language models are increasingly used as tools for reading, summarizing, drafting, and evaluating text. Because many professional decisions are mediated by language—including legal decisions—there is growing interest in whether such models can be used as decision-support systems. In legal contexts, this question is not only about whether a model can generate fluent or plausible explanations, but whether its judgments are reliable and stable. In particular, a system used to support legal decisions should not change its conclusions simply because information is presented in a different order or because an additional assessment step is introduced. Sensitivity to such procedural features is especially problematic in legal settings, where consistency, fairness, and justification are central normative requirements.

### 1.1. Bias and Rationality in Large Language Models

An emerging literature has examined whether large language models exhibit biases similar to those observed in human judgment, and whether such patterns change across model generations. Some studies suggest that models do reproduce classic human decision-making biases. For example, Suri et al. (2023) showed that GPT-3.5 displayed several well-known heuristics from the human literature, including anchoring, representativeness, availability, framing, and endowment effects, with model outputs systematically influenced by normatively irrelevant contextual cues.

Extending this line of work, Yax et al. (2024) compared human reasoning with that of multiple GPT models across a range of cognitive psychology tasks. Earlier models often produced intuitive but incorrect answers that closely resembled common human errors, whereas more recent models—such as GPT-4—were generally more accurate and showed reduced susceptibility to classic biases, like the conjunction fallacy. This pattern suggests that improvements in model capabilities can reduce some forms of human-like bias.

At the same time, other work cautions against interpreting all apparent errors in large language models as human-like cognitive biases. Macmillan-Scott and Musolesi (2024), for instance, found that many model failures across a variety of reasoning tasks reflected inconsistency, illogical reasoning, or sensitivity to prompt wording, rather than the fairly stable, systematic biases of the kind observed in humans. In some cases, the same model produced different answers to the same problem across runs, alternating between correct and incorrect reasoning.

Taken together, these findings point to a mixed picture. Large language models can sometimes mirror biases observed in human judgment, but they can also show sensitivity to procedurally irrelevant factors in ways that do not neatly align with human patterns. From an applied perspective—for example, in legal contexts—one central concern is whether judgments remain stable when informational content is held constant. Concerns about bias in legal decision-making are not limited to algorithmic systems; work in forensic and legal psychology has long documented systematic biases in expert judgments that often influence legal outcomes (Buongiorno et al., 2025). The present study addresses this issue by examining procedural sensitivity in two GPT models (GPT-4o and GPT-5.2) and comparing their judgments with those of human participants in a legal decision-making task.

### 1.2. Bias Mitigation and Prompt Engineering

If procedural biases do arise in large language models, a natural question is whether they can be reduced. Alongside efforts to identify biases in large language models, recent work has examined whether such biases can be mitigated through changes to how models are prompted. One widely studied approach is prompt engineering, which involves modifying the wording or structure of instructions to influence model behavior. Techniques such as chain-of-thought prompting have been shown to improve performance on some reasoning tasks and, in certain cases, reduce biased responses (Wei et al., 2023). Other studies suggest that more structured or reflective prompts can attenuate specific biases in large language models, though effects vary across tasks and models (Koralus & Wang-Maścianica, 2023; Sumita et al., 2024). At the same time, evidence indicates that mitigation strategies effective for humans do not always translate to large language models (Yax et al., 2024). This raises the question of whether prompt engineering can meaningfully reduce biases in judgment tasks where there is an expectation of normative behavior, such as in legal decision-making.

There is a wide range of potential biases that can be examined when exploring these issues in large language models. In the present study, we follow Wojciechowski et al. (2025), who focused on two specific biases: order effects and evaluation biases. Both biases (explained below) have been argued to be particularly relevant in legal decision-making, which motivates their investigation here. An additional advantage of grounding the present investigation in a prior human experimental study is that it allows us to adopt the same procedure and methodology.

### 1.3. Order Effects

One source of potential judgment bias that is especially relevant to legal decision-making is order effects. Order effects refer to changes in a judgment that arise from the sequence in which information is presented, even when the informational content remains unchanged.

Order effects have been documented across many domains of judgment. For example, Moore (2002) showed that simply changing the order in which people were asked about the honesty of political figures (Clinton and Gore) substantially shifted response patterns, despite identical questions being asked. Similarly, in a medical context, Bergus et al. (1998) found that physicians’ diagnostic judgments differed depending on whether lab results or physical examination findings were presented first, even though both pieces of information were always provided. These findings illustrate that the sequence of information alone—independent of content—can systematically influence judgment in applied decision-making contexts.

In legal psychology, order effects have been studied extensively in contexts such as evidence presentation and juror decision-making (e.g., Trueblood & Busemeyer, 2011). For example, work by Pennington and Hastie (1992) shows that the organization of evidence can shape how decision-makers construct narratives about a case, making judgments sensitive to the structure and order in which information is encountered.

Within this literature, particular attention has been given to recency effects, whereby evidence presented later in the sequence exerts a disproportionate influence on final judgments. For example, Costabile and Klein (2005) showed that incriminating evidence was more likely to lead to guilty verdicts when it was presented late rather than early, even when jurors were instructed to withhold judgment until all evidence had been presented. Costabile and Klein further suggested that this effect was mediated by memory, with later-presented evidence being more accessible at the time of judgment. Similarly, McKenzie et al. (2002) reported a consistent recency effect in jury decision-making: verdicts tended to shift towards guilt when the final evidence suggested guilt, and towards innocence when exonerating information appeared last. More directly related to the present study, Wojciechowski et al. (2025) demonstrated strong recency effects in a legal judgment task using simplified criminal cases, showing that final guilt ratings were systematically higher when incriminating evidence was presented last. Such effects are normatively problematic because they show that judgments about guilt can change due to presentation order, rather than differences in evidential content.

Given the importance of order effects in human legal judgment, a natural question is whether similar sensitivities appear in large language models. Order sensitivity has indeed been examined in large language models, although not all reported ‘order effects’ refer to the same phenomenon. For example, several studies document positional biases in multiple-choice tasks, where model responses depend on the position of answer options rather than their content (Pezeshkpour & Hruschka, 2023; Ranaldi & Zanzotto, 2023).

Following Wojciechowski et al. (2025), the present study focuses instead on order effects in evaluative judgments arising from differences in the sequence of evidence presentation, as studied in legal decision-making. Unlike positional biases at the response stage, evidence-order effects concern how information is integrated over time. This distinction is critical for assessing the use of large language models in legal decision-support contexts.

### 1.4. Evaluation Biases

An additional class of bias examined by Wojciechowski et al. (2025) concerns evaluation biases. In general, beyond the order in which information is presented, judgments can also be shaped by when evaluations are made. Evaluation biases (there is not a single kind) concern cases in which the act of making an intermediate judgment changes a later judgment, even when the underlying information remains the same.

A particular evaluation bias was demonstrated by White et al. (2014) with made-up advertisements and later examined in more detail by White et al. (2020). In their studies, participants were exposed to pairs of stimuli with opposing valence (e.g., one negative and one positive). Participants always evaluated the second stimulus, but in some conditions they also evaluated the first. Across materials and task variants, a consistent pattern emerged: when participants made an intermediate evaluation, their subsequent judgments became more extreme. For example, positive stimuli were rated more positively when participants had also evaluated the preceding stimulus, compared to when they evaluated the final stimulus alone. Importantly, this shift occurred despite no change in the information available at the time of the final judgment. The bias therefore reflects an effect of the evaluation procedure itself, rather than differences in evidence.

Wojciechowski et al. (2025) extended this paradigm to a legal judgment task, showing that requiring intermediate guilt assessments can systematically influence final guilt ratings. If large language models are used in workflows that involve staged or incremental evaluations—such as preliminary screenings, early risk assessments, or stepwise reviews—evaluation-related biases become practically relevant, as the structure of the decision process alone may shape outcomes.

### 1.5. The Present Study

Despite growing interest in the use of large language models in professional decision-making, it remains unclear whether such models exhibit the same procedural biases documented in human legal judgment, and whether commonly proposed mitigation strategies can reduce these effects.

The present study tests whether two GPT models exhibit these two forms of procedural sensitivity in a legal decision-making paradigm and whether prompt engineering can reduce them. We closely follow the experimental design from Wojciechowski et al. (2025), in which decision-makers read simplified real criminal cases before rating the defendant’s guilt. The design manipulates (i) the order in which incriminating and exonerating evidence is presented and (ii) whether an intermediate judgment is required before the final judgment. We compare model behavior to human data from the original study. We also examine prompt engineering manipulations to reduce large language model putative bias, motivated by contemporary guidance on how to shape large language model reasoning.

More specifically, the study has three objectives. First, we test whether GPT-4o and GPT-5.2 exhibit an order effect in final guilt ratings, when evidential content is held constant but evidence order is reversed. Second, we test whether requiring an intermediate judgment produces an evaluation bias of some sort in these models and whether the specific evaluation bias matches the one observed in White et al. (2020) and Wojciechowski et al. (2025). Third, we test whether prompt engineering strategies that emphasize legal expertise, careful deliberation, or explicit justification reduce the magnitude of any observed biases.

The present work provides (i) a direct comparison of human and large language model judgments under matched legal procedures, (ii) a characterization of order- and evaluation-related biases in GPT models, and (iii) an empirical assessment of prompt engineering as a bias-mitigation strategy in legal-style judgment tasks.

By focusing on procedural manipulations that are normatively irrelevant but practically common in legal decision-making, this work aims to contribute to a more realistic assessment of whether large language models can be treated as stable decision-support systems in high-stakes judgment contexts.

## 2. Materials and Methods

The aim of this study was to examine the presence of order effects and evaluation bias in large language models, specifically GPT-4o and GPT-5.2, and to compare their responses to those of human participants. To this end, we adapted materials from a study originally conducted with human participants (Wojciechowski et al., 2025). The experimental procedure was followed as closely as possible, with necessary adaptations reflecting the fact that a large language model is not a human participant. In addition to comparing model responses with the original human data, we extended the design by introducing several prompt engineering conditions to explore whether such strategies could reduce bias in the models’ judgments.

### 2.1. Participants

The human data analyzed in the present study were drawn from the experiment reported by Wojciechowski et al. (2025). The sample comprised legal professionals (judges, prosecutors, and defense attorneys) as well as participants without a legal background, all recruited in Poland. Participants varied in age, gender, and professional experience, with legal professionals spanning a wide range of years in practice. Participation was voluntary and uncompensated, and ethical approval was obtained by the original authors.

A full description of participant characteristics, recruitment procedures, and ethical considerations is provided in the original publication.

### 2.2. Models

The models employed in the experiment were GPT-4o-2024-05-13 and GPT-5.2-2025-12-11, two specific releases from OpenAI’s GPT-4 and GPT-5 model families. All interactions with the models were conducted via OpenAI’s Application Programming Interface (API) using Python (v3.9.12). The API uses a structured message format involving three roles: system, user, and assistant. The system message provides general task instructions and contextual guidance. The user message contains the materials and questions presented to the model. The assistant message corresponds to the model’s generated response based on the provided inputs.

To control response variability, the temperature parameter was set to 1 for the primary experimental runs. The temperature parameter controls how variable the model’s responses are, with lower values producing more deterministic outputs and higher values producing greater variability. A value of 1 corresponds to OpenAI’s default setting and was chosen to reflect standard usage while keeping response variability constant across conditions. All other model parameters were left at their default settings.

As a robustness check, additional analyses were conducted at a lower temperature (*T* = 0.2). Details of these analyses are reported below.

### 2.3. Materials and Design

The materials consisted of six real (but simplified) Polish criminal cases obtained from various District Courts and Appellate Courts in Poland, concerning crimes committed between 2000 and 2015 (see Wojciechowski & Pothos, 2018 for details on case selection). The same cases were used in the human experiment reported in Wojciechowski et al. (2025). For the present study, the case materials were translated into English, as all interactions with GPT were conducted in English.

The translation was carried out by one of the co-authors, who also contributed to the original study and who is a native Polish speaker fluent in English. The translation aimed to preserve the original meaning, structure, and balance between incriminating and exonerating information, and the same English translations were used across all model conditions. The full English case descriptions used in the experiment can be found in the original publication.

Each case followed an identical structure. A preamble introduced the charges against the defendant, followed by two evidentiary parts: an incriminating part suggesting the defendant’s guilt (Part G) and an exonerating part suggesting innocence (Part I). The order of these two parts (G–I vs. I–G) constituted one experimental manipulation. The model’s task was to assess the probability of the defendant’s guilt on a scale from 1 (definitely innocent) to 10 (definitely guilty). This manipulation allowed us to test for an order effect, defined as a systematic influence of evidence order on final guilt judgments.

A second manipulation concerned the rating procedure, as in the design of Wojciechowski et al. (2025). In the single-rating condition, the model provided one final guilt judgment after reading the full case (preamble, Part 1, and Part 2). In the double-rating condition, the model first provided an intermediate judgment after reading the preamble and the first evidentiary part, followed by a final judgment after reading the second part. This manipulation was intended to test for an evaluation bias—namely, whether the requirement to provide an intermediate judgment affects the final assessment, despite identical informational content.

The experiment thus followed a 2 (order: G–I vs. I–G) × 2 (rating condition: single vs. double) × 6 (case) design. All factors were treated as between-trial variables: each trial consisted of a single case presented in one order and one rating condition, and trials were independent, with no information carried over from one trial to the next.

**Figure 1:**
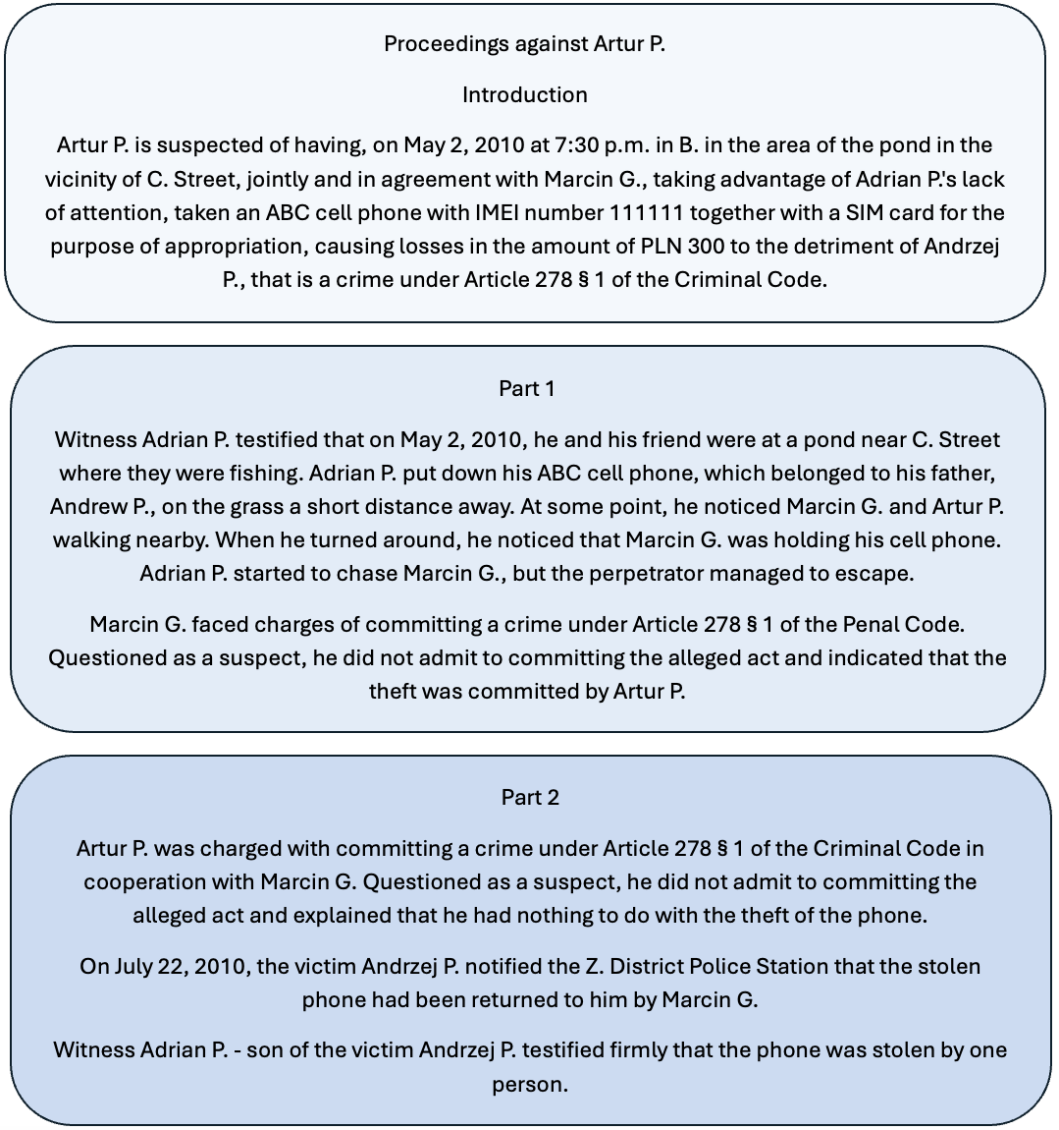
Example of a criminal case description presented in the G–I order. All cases shared the same structure: a preamble followed by two evidentiary parts presented in either G–I or I–G order.

An example case is shown in Figure 1. The top box presents the preamble. The second box shows Part 1, which in this example is incriminating (G), and the third box shows Part 2, which is exonerating (I), corresponding to the G–I order. In the I–G condition, the order of these two parts is reversed.

A total of N=720 trials were conducted for each model and prompt engineering condition. This number was chosen to mirror the human experiment, which involved 120 participants who collectively provided 715 final judgments (approximately 120 per case). Accordingly, 720 trials were run to ensure a comparable number of final ratings per case. Because trial conditions were randomly assigned in the model experiments, the exact number of trials per case, order, and rating condition varied slightly.

### 2.4. Procedure

#### 2.4.1. Baseline Procedure

At the beginning of each trial, the experimental conditions—namely, one criminal case (out of six), one presentation order (G–I or I–G), and one rating condition (single or double)—were randomly assigned from their respective sets of options (e.g., case = Artur, order = G–I, rating condition = double).

The model (GPT-4o-2024-05-13 or GPT-5.2-2025-12-11, depending on the experimental run) was presented with general task instructions delivered via the system prompt. These instructions were designed to closely resemble those given to human participants in the original study by Wojciechowski et al. (2025). Note that in the subsequent prompt engineering manipulation (introduced in the following section), it is these instructions that were modified with the aim of reducing bias. The full texts of the human instructions—including the original Polish materials and their English translations—are provided in the original publication.

Note that human participants were not given any role-based framing, whereas GPT was. Indeed, in the baseline condition, PE 0 (and two of the prompt engineering conditions), GPT was addressed using the default system prompt (“You are a helpful assistant”), alongside the task instructions. This prompt was included because it reflects the default configuration under which GPT is commonly used in practice, and thus provides a realistic baseline for common usage. The same prompt was not used for human participants because the instructions given to humans implicitly convey an expectation that they should respond in a cooperative and task-appropriate manner (cf. conversational implicatures; Grice, 1975), whereas such expectations must be stated explicitly when interacting with language models. While we do not expect this difference to substantially affect comparability with human instructions, it does constitute a potential source of procedural asymmetry.

**Figure 2:**
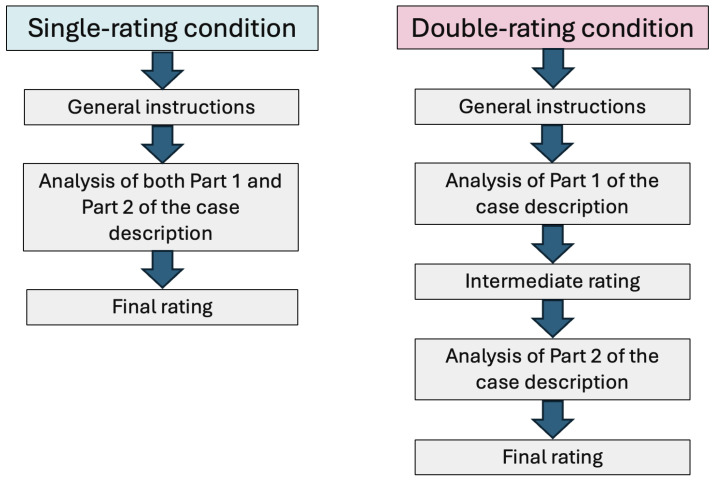
Overview of the experimental procedure in the single- and double-rating conditions. In the single-rating condition (**left**), the model received general instructions, analyzed both Part 1 and Part 2 of the case description, and provided a single final guilt rating. In the double-rating condition (**right**), the model gave an intermediate rating after analyzing Part 1, followed by a final rating after analyzing Part 2.

Following the general instructions, the model was presented with a criminal case description and asked to evaluate the guilt of the defendant on a scale from 1 to 10, with 1 indicating “definitely innocent” and 10 indicating “definitely guilty”. In the double-rating condition, the model additionally provided an intermediate rating immediately after reading the first part of the case. A general overview of the experimental procedure is provided in Figure 2.

Following presentation of the case description, the model was prompted to provide a guilt judgment using the following question:

Evaluate the probability that [defendant’s name] committed the alleged crime, using the following scale and assuming that:

1—definitely innocent       10—definitely guilty

You are being asked to respond on a 1–10 scale, as a multiple-choice response. Do not give any other rating. Do not put any text before or after your rating.

The same question was used to elicit both intermediate and final ratings.

Each trial was conducted as an independent session. No conversational history or responses were carried over between trials, and no persistent session state was used across API calls. The only exception occurred in the double-rating condition, where context was intentionally maintained across the two ratings within a single trial as part of the experimental manipulation. Consequently, ratings across trials could be treated as independent observations.

#### 2.4.2. Prompt Engineering Procedure

**Figure 3:**
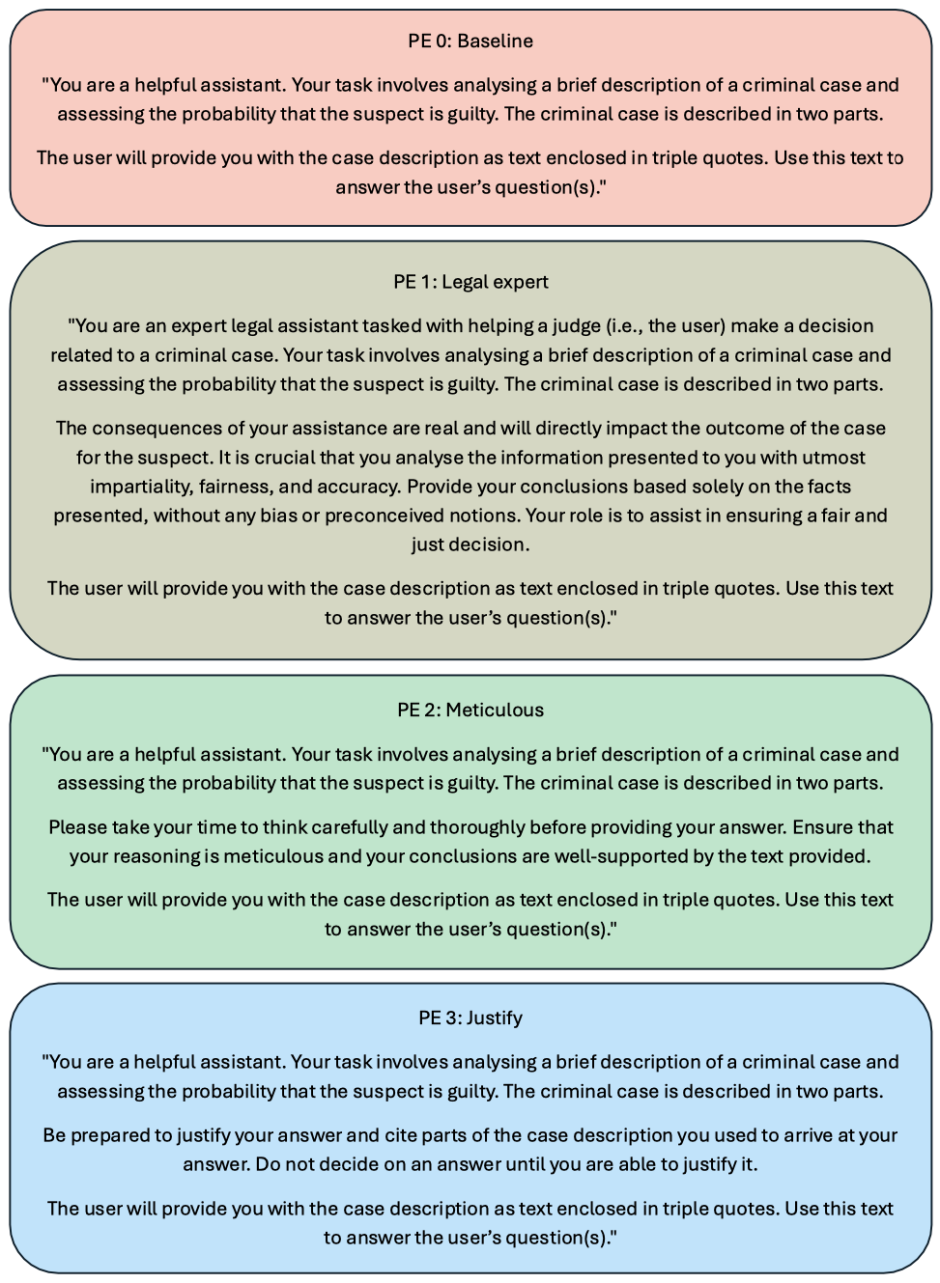
The four system prompts used across the prompt engineering conditions. PE0 served as the baseline, while PE1 (Legal expert), PE2 (Meticulous), and PE3 (Justify) implemented different prompt engineering strategies inspired by guidance provided in OpenAI’s documentation.

In addition to testing the effects of order and rating condition on the models’ guilt ratings, we examined whether modifying the general instructions provided to the model influenced its responses. To do so, we created three alternative versions of the system prompt, denoted PE1, PE2, and PE3 (‘PE’ standing for prompt engineering), alongside the baseline prompt (PE0), for a total of four conditions. These are illustrated in Figure 3.

Prompt engineering refers to the process of designing and refining the inputs given to a language model in order to guide its responses. In the present study, prompt engineering was used to explore whether changes in task framing could reduce the models’ susceptibility to order and evaluation biases.

OpenAI’s documentation on prompt engineering outlines several broad strategies for influencing model behavior. Drawing on this guidance, we selected some prompting strategies that appeared particularly relevant to decision-making tasks. Because the range of possible prompt engineering strategies is effectively unbounded, the examples examined here should be understood as illustrative rather than as a comprehensive evaluation.

1. *Adopting a persona* (part of the broader strategy *Write clear instructions*). This approach involves instructing the model to respond as if it were a specific individual or professional, such as a domain expert. In PE1, the model was instructed to adopt the persona of a legal expert. The goal was to encourage responses grounded in legal reasoning, under the assumption that a legal professional might be less prone to certain cognitive biases. The prompt also emphasized the importance of impartiality, fairness, and accuracy in decision-making.
2. *Encouraging deliberate reasoning*. This strategy aims to reduce overly hasty responses by prompting the model to reflect more carefully before answering. We tested two variations of this approach. In PE2, the model was explicitly instructed to take its time and to reason carefully and thoroughly before providing a judgment. In PE3, the model was additionally asked to be prepared to justify its final judgment by referring to specific elements of the case description.

Within each prompt engineering condition, only the system prompt was modified. All other aspects of the experimental procedure were held constant across conditions. The same set of prompt engineering conditions (PE0–PE3) was applied to both GPT-4o and GPT-5.2. We note that PE0 closely mirrored the instructions given to human participants in the original study and did not incorporate any explicit bias-mitigation strategies. As such, it provided a natural negative control reference point against which the effects of the prompt engineering interventions (PE1–PE3) could be evaluated.

Note that we did not employ chain-of-thought prompting or other instructions that explicitly require the model to generate intermediate reasoning steps. Because the design already manipulates whether an intermediate judgment is required, such prompts would have altered the task structure and confounded the mechanism under investigation.

The prompt engineering manipulations examined here were intended as representative examples of commonly recommended prompting strategies rather than a comprehensive exploration of prompt design. A broader factorial investigation of prompting strategies could reveal additional approaches that influence model behavior in judgment tasks.

## 3. Results

Our analyses addressed three primary questions. First, do large language models exhibit order effects and evaluation-related biases comparable to those observed in humans? Second, do such biases persist across model generations? Third, can prompt engineering mitigate these effects? To answer these questions, we analyzed data from human participants, GPT-4o, and GPT-5.2 using linear modeling approaches.

All analyses reported in this section focus on final guilt ratings, defined as the single rating provided in the single-rating condition and the second (final) rating provided in the double-rating condition. Baseline results are reported first for the PE0 condition, followed by analyses of prompt engineering effects.

### 3.1. Baseline Results

Baseline analyses focused on the PE0 condition (i.e., instructions matched to those given to human participants). Baseline effects were examined separately for each agent (human participants, GPT-4o, and GPT-5.2) before turning to a combined model.

For both GPT models, final guilt ratings were analyzed using linear models including order of presentation (G–I vs. I–G), rating condition (single vs. double), and their interaction as predictors, with case included as a fixed effect.

For human participants, we report the results from the original study on which the present design is based. In that study, the analysis followed a richer model specification that additionally included role and its interactions with the other predictors, as well as interactions involving case, and a random intercept for participant ID (with no random slopes).

In addition, a combined baseline model was estimated on the present data to directly compare humans and GPT models. This model included subject (human, GPT-4o, GPT-5.2) as a fixed effect and its interactions with order and rating condition, with case retained as a fixed effect.

#### 3.1.1. GPT-4o

We first examined baseline results for GPT-4o in the PE0 condition, in which the general instructions closely mirrored those used in the human experiment by Wojciechowski et al. (2025). GPT-4o produced 720 final guilt ratings in this condition. Descriptive statistics by order and rating condition are shown in Table 1.

To analyze the GPT-4o data, we fitted a linear model of the form

rating∼order×ratingcondition+case.

The linear model revealed a robust main effect of evidence order, with significantly higher final guilt ratings in the I–G condition than in the G–I condition, F(1,711) = 424.76, p<0.001, indicating a clear recency effect whereby incriminating information presented last increased perceived guilt. This result shows an order effect.

There was also a significant main effect of rating condition, F(1,711) = 181.11, p<0.001, such that final guilt ratings were higher in the double-rating condition than in the single-rating condition.

There was also a strong main effect of case, F(5,711)=600.98, p<0.001, indicating substantial variation in baseline guilt ratings across the six criminal cases.

The interaction between order and rating condition was also statistically significant, F(1,711)=43.75, p<0.001. This interaction reflected that the effect of the rating condition differed in magnitude across evidence orders: the increase in guilt ratings associated with the double-rating condition was larger in the I–G condition than in the G–I condition. Importantly, however, the direction of the effect did not reverse across orders. In both presentation orders, requiring an intermediate judgment increased final guilt ratings. This result shows an evaluation bias, but not of the same kind as the one observed by Wojciechowski et al. (2025). We refer to this evaluation bias as a *measurement bias*, which concerns this GPT-specific behavior whereby the act of providing an intermediate judgment systematically inflated GPT-4o’s final guilt assessments, despite identical informational content.

#### 3.1.2. GPT-5.2

Baseline analyses for GPT-5.2 were conducted using the same design and statistical model as for GPT-4o. GPT-5.2 produced 720 final guilt ratings in the PE0 condition. Descriptive statistics by order and rating condition are shown in Table 2.

The linear model fitted to the GPT-5.2 data was identical to the model used for the GPT-4o analysis.

The linear model revealed a significant main effect of evidence order, F(1,711)=71.24, p<0.001, with higher final guilt ratings in the I–G condition than in the G–I condition. This indicates that a recency effect persists in GPT-5.2, although note that its magnitude is notably smaller than that observed for GPT-4o.

GPT-5.2 showed a significant main effect of rating condition, F(1,711)=8.43, p=0.0038. As above, the magnitude of this effect was markedly reduced relative to that observed in GPT-4o, indicating a weaker sensitivity to the rating procedure in GPT-5.2.

There was also a significant main effect of case, F(5,711)=1780.60, p<0.001.

Regarding evaluation biases, there was a significant interaction between evidence order and rating condition, F(1,711)=39.83, p<0.001. This interaction reflected an order-dependent effect of the rating procedure. In the G–I condition, final guilt ratings were similar in the single- and double-rating conditions. In contrast, in the I–G condition, requiring an intermediate judgment led to higher final guilt ratings than in the single-rating condition. Thus, GPT-5.2 did not exhibit the measurement bias observed in GPT-4o. Instead, the influence of the rating procedure was somewhat closer to the evaluation bias observed with human participants. Note that the magnitude of these effects indicates a partial attenuation of these kind of biases in GPT-5.2 relative to GPT-4o.

Finally, as a robustness check, we repeated the baseline condition (PE0) at a lower temperature (T=0.2) for both GPT-4o and GPT-5.2 using the same experimental procedure (N=720 trials). Model judgments aggregated by case, order, and rating condition were highly correlated across temperature settings (GPT-4o: r=0.996; GPT-5.2: r=0.989), indicating that the observed bias patterns are not driven by temperature-dependent stochasticity.

#### 3.1.3. Human Participants

For completeness, we review the analyses for human participants reported in Wojciechowski et al. (2025). As in the GPT models, final guilt ratings were analyzed as a function of evidence order and rating condition. In the human data, these predictors were additionally allowed to interact with case and participant role (Attorney, Judge, Prosecutor, Lay Person), reflecting the differing objectives between the human experimental work and the present work. The model also included a random intercept for participant identity. Descriptive statistics by order and rating condition are shown in Table 3.

Specifically, in the original study, human data were analyzed using a linear mixed-effects model of the form

rating∼order×ratingcondition×role×case+(1∣id).

Human participants exhibited a main effect of evidence order, with significantly higher guilt ratings in the I–G condition than in the G–I condition, F(1,686)=54.40, p<0.001, indicating a clear recency effect.

There was no main effect of rating condition, F(1,644)=3.37, p=0.067, indicating that providing an intermediate judgment did not uniformly increase or decrease final guilt ratings.

However, the interaction between order and rating condition was statistically significant, F(1,709)=8.53, p=0.004. This interaction indicates an evaluation bias. The pattern of means was consistent with the evaluation bias described by White et al. (2020), with intermediate judgments amplifying the influence of the most recently presented information.

There was also a significant main effect of case, F(5,589)=40.68, p<0.001.

#### 3.1.4. Comparison Across Humans and Models

To compare response patterns across human participants, GPT-4o, and GPT-5.2, we estimated a combined baseline model including subject type and its interactions with evidence order and rating condition, with case included as a fixed effect.

In this combined analysis, participant role was not included because it is only defined for human data and has no analog in model responses. Likewise, the random intercept for participant identity was omitted, as GPT responses are not nested within individual subjects in the same way as human judgments. Including these terms would therefore introduce structural asymmetries across subject types.

Note that interactions involving case were also not included in the GPT or combined models. In the human analysis, case interactions were used to account for heterogeneity across specific legal scenarios. By contrast, the GPT analyses focus on identifying general procedural biases across cases rather than modeling case-specific variation. Including higher-order interactions with case in these models would substantially increase complexity, without directly addressing the primary research questions.

The linear model fitted to the combined data was of the form

rating∼order×ratingcondition×subject+case.

We first consider some results in the combined dataset, irrespective of agents. A main effect of subject was observed, F(2,2138)=7.74, p<0.001, indicating overall differences in mean guilt ratings across agents. Across agents, there was a main effect of evidence order, F(1,2138)=205.09, p<0.001, indicating a general recency effect whereby guilt ratings increased when incriminating evidence was presented last. Across agents, there was also a statistically significant main effect of rating condition, F(1,2138)=20.76, p<0.001, indicating overall differences between single- and double-rating procedures. Finally, there was also a strong main effect of case, F(5,2138)=493.08, p<0.001. Case-level plots for humans, GPT-4o, and GPT-5.2 are reported in Appendix A.

**Figure 4:**
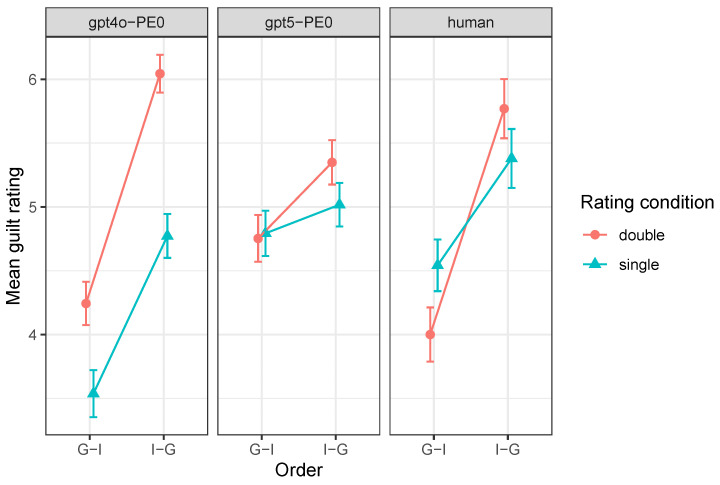
Mean final guilt ratings by order and rating condition in the PE0 (baseline) condition for GPT-4o and GPT-5.2, alongside human data. Error bars represent ±1 standard error of the mean. Lines connect means for visual clarity only and do not represent within-subject or within-trial conditions.

More pertinently, the magnitude of the order effect differed significantly between agents (order × subject), F(2,2138)=17.31, p<0.001. Inspection of the means in Figure 4 suggests stronger order effects for GPT-4o and human participants, with a markedly reduced effect in GPT-5.2.

The influence of the rating procedure also differed across agents, as reflected in a significant rating condition × subject interaction, F(2,2138)=12.44, p<0.001. Inspection of the means in Figure 4 suggests that GPT-4o showed a strong uniform increase in guilt ratings following intermediate judgments, GPT-5.2 a weaker and more order-dependent effect, and humans a qualitatively different pattern consistent with an order-dependent reversal.

The three-way interaction between order, rating condition, and subject was not significant, F(2,2138)=1.05, p=0.35.

Figure 4 illustrates these patterns.

### 3.2. Prompt Engineering Results

We next examined whether prompt engineering could reduce order effects or evaluation biases in model judgments. Prompt engineering analyses were conducted separately for GPT-4o and GPT-5.2 using four system-prompt variants: the baseline prompt (PE0) and three alternative prompts (PE1, PE2, and PE3). For each prompt condition, 720 trials were run, yielding 2880 final ratings per model. All analyses used linear models including order, rating condition, prompt engineering condition, and their interactions, with case included as a fixed effect.

We did not directly compare prompt-engineered model conditions with human judgments, because such comparisons would be difficult to interpret and would increase statistical complexity. Prompt engineering manipulations were applied only to the models, whereas human participants received only the baseline instructions, meaning that any observed differences would conflate agent type with instructional framing.

#### 3.2.1. GPT-4o

The linear model fitted to the GPT-4o prompt engineering (PE) data was of the form

rating∼order×ratingcondition×PE+case.

Across prompt engineering conditions, GPT-4o showed a statistically significant order effect: final guilt ratings were higher when incriminating evidence was presented last (I–G) compared to when it was presented first (G–I), F(1,2859)=1355.74, p<0.001. However, the magnitude of the order effect differed across prompt engineering conditions, as reflected in a significant order × prompt interaction, F(3,2859)=3.11, p=0.026.

To identify which prompt variants differed from the baseline condition (PE0), we conducted post hoc contrasts comparing the order effect (I–G minus G–I) within each prompt engineering condition to the corresponding effect under PE0. These contrasts revealed that PE2 significantly attenuated the order effect relative to PE0 (Δ=−0.32, SE=0.11, t=−2.99, p=0.003). By contrast, neither PE1 (Δ=−0.19, p=0.075) nor PE3 (Δ=−0.16, p=0.12) differed reliably from the baseline condition. It is worth noting that although PE2 reduced the magnitude of the order effect, a statistically significant order effect remained present under all prompt engineering conditions, indicating that prompt engineering did not eliminate sensitivity to evidence order in GPT-4o.

GPT-4o also showed a statistically significant main effect of rating condition, F(1,2859)=427.95, p<0.001, across prompt engineering variants.

**Figure 5:**
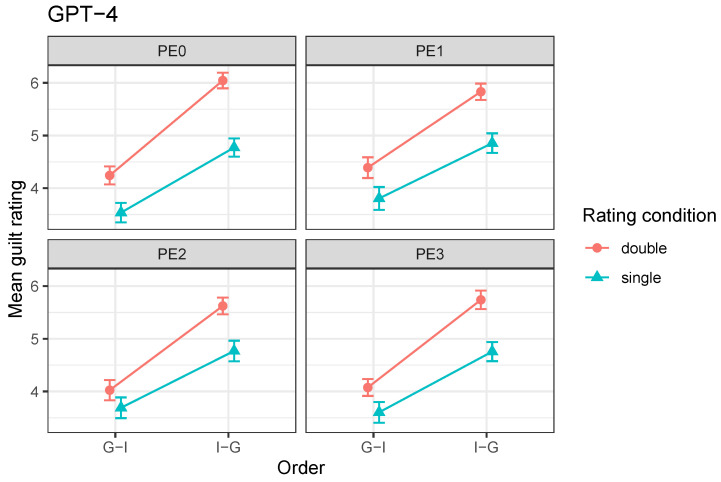
Mean final guilt ratings by order and rating condition across prompt engineering conditions for GPT-4o. Error bars represent ±1 standard error of the mean. Lines connect means for visual clarity only and do not represent within-trial changes.

Neither the rating × prompt interaction, F(3,2859)=1.23, p=0.30, nor the three-way interaction between order, rating condition, and prompt engineering, F(3,2859)=1.55, p=0.20, reached statistical significance. Accordingly, prompt engineering did not reliably modify the specific evaluation bias observed here—namely, the measurement bias. As illustrated in Figure 5, across all prompt variants, final guilt ratings remained higher in the double-rating condition than in the single-rating condition, regardless of evidence order.

Finally, prompt engineering condition had a significant main effect on overall guilt ratings, F(3,2859)=5.59, p<0.001.

#### 3.2.2. GPT-5.2

The linear model fitted to the GPT-5.2 PE data was identical to the model used for the GPT-4o PE analysis.

Across all prompt engineering conditions, GPT-5.2 showed a statistically significant order effect: final guilt ratings were higher when incriminating evidence was presented last (I–G) compared to when it was presented first (G–I), F(1,2859)=574.40, p<0.001. The magnitude of the order effect varied across prompt engineering conditions, as reflected in a significant order × prompt interaction, F(3,2859)=14.76, p<0.001.

Post hoc contrasts comparing the size of the order effect (I–G minus G–I) under each prompt engineering condition to the baseline condition (PE0) revealed no reliable differences for PE1 (Δ=0.03, p=0.62) or PE2 (Δ=0.07, p=0.28). In contrast, PE3 significantly *increased* the magnitude of the order effect relative to PE0 (Δ=0.39, SE=0.07, t=5.91, p<0.0001), indicating an amplification rather than an attenuation of order sensitivity under this prompt. A statistically significant order effect remained present under all prompt engineering conditions, so, as above, prompt engineering did not eliminate order effects in GPT-5.2.

GPT-5.2 further showed a statistically significant main effect of rating condition, F(1,2859)=87.87, p<0.001, indicating differences between single- and double-rating conditions across prompt engineering variants. In addition, a significant rating condition × prompt interaction was observed, F(3,2859)=8.12, p<0.001. This interaction with the rating factor suggests that prompt engineering manipulations might affect either the measurement bias or the evaluation bias from Wojciechowski et al. (2025) or something else. So, in post hoc contrasts we explored this effect of rating condition using a variable corresponding to the evaluation bias defined by Wojciechowski et al. (2025) (judgment in the double-rating condition minus in the single-rating one), under each prompt engineering condition. Comparisons against the baseline condition (PE0) revealed no reliable difference for PE2 (Δ=−0.06, p=0.36). In contrast, both PE1 (Δ=0.16, p=0.018) and PE3 (Δ=0.23, p<0.001) significantly increased the magnitude of the evaluation bias relative to PE0, indicating an amplification rather than an attenuation of the measurement bias under these prompt variants. In other words, the difference in final rating between double- and single-rating conditions was larger under PE1 and PE3 than under the baseline condition.

**Figure 6:**
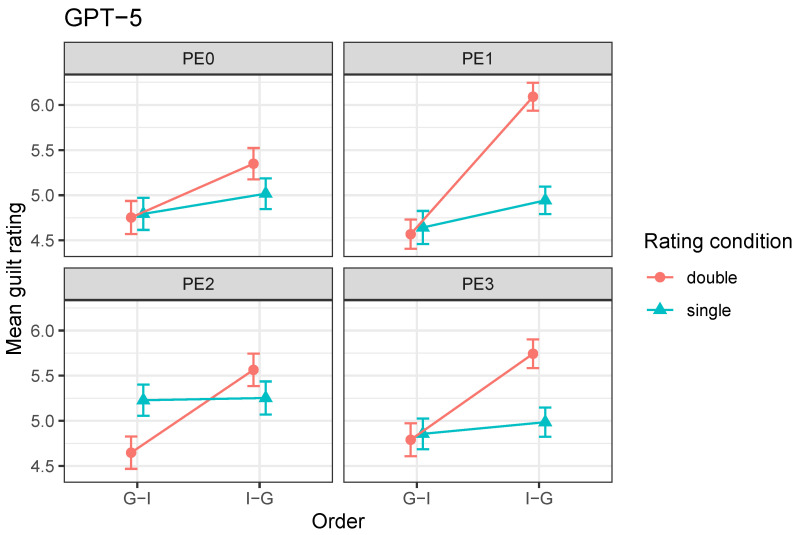
Mean final guilt ratings by order and rating condition across prompt engineering conditions for GPT-5.2. Error bars represent ±1 standard error of the mean. Lines connect means for visual clarity only and do not represent within-trial changes.

Visual inspection of the means (Figure 6) suggests that under PE2, the effect of the rating condition became order-dependent. Specifically, the difference between double- and single-rating conditions is reversed across evidence orders, with higher ratings in the single-rating condition for G–I but higher ratings in the double-rating condition for I–G. This pattern resembles the evaluation bias of Wojciechowski et al. (2025) and contrasts with the measurement bias observed in the baseline condition (PE0). In contrast, under PE1 and PE3, the effect of rating condition remained asymmetric across orders: differences between double and single ratings were pronounced in the I–G condition but minimal in the G–I condition, similar to the baseline pattern. In line with this observation, the three-way interaction between order, rating condition, and prompt engineering almost reached statistical significance, F(3,2859)=2.58, p=0.052.

Finally, prompt engineering condition had a significant main effect on overall guilt ratings, F(3,2859)=14.05, p<0.001.

## 4. Discussion

The present study examined whether GPT models exhibit cognitive biases similar to those observed in human legal decision-making, with a focus on order effects and evaluation biases. Using a controlled judgment task adapted from prior human research, we compared human participants with GPT-4o and GPT-5.2 and assessed whether prompt engineering could reduce biased responses.

Three main conclusions emerged. First, humans, GPT-4o, and GPT-5.2 all showed order effects in the baseline condition: guilt ratings were higher when incriminating evidence was presented last rather than first. Although this effect was substantially smaller in GPT-5.2 than in GPT-4o, it did not disappear. From a normative perspective, such order sensitivity is problematic, as a rational decision-maker should reach the same conclusion regardless of the sequence in which identical evidence is presented. Under standard Bayesian principles, when the same pieces of evidence are incorporated into a belief update, the resulting posterior probability should be independent of the order in which they are considered (i.e., P(A)P(B|A)=P(B)P(A|B)). This property holds regardless of the content of the propositions involved and therefore applies equally to legal evidence. If final judgments differ depending on presentation order, this implies that the weighting of evidence depends on its position in the sequence rather than solely on its evidential content.

The presence of order effects in both GPT-4o and GPT-5.2 suggests that GPT models, like humans, are sensitive to the sequential structure of information. It very much appears that information presented later receives greater weight when forming a judgment. This pattern may reflect regularities in natural language, where later statements often function as updates or conclusions. Alternatively, it might relate to decision strategies, whereby a person might reserve the most compelling argument or evidence for last (taking advantage of memory effects to make such evidence more salient). It is interesting that order effects persist across model generations.

Second, the influence of intermediate judgments differed between humans and models, and across model generations. In human participants, as Wojciechowski et al. (2025) reported, the requirement to provide an intermediate judgment produced a pattern consistent with the classic evaluation bias reported in prior work, whereby intermediate judgments tend to amplify the influence of the most recently presented information.

GPT-4o, however, showed a qualitatively different pattern. Rather than exhibiting opposite shifts across evidence orders, final guilt ratings were consistently higher when an intermediate judgment was required, regardless of order. We refer to this pattern as a measurement bias. In GPT-4o, this bias was present for both orders and was stronger when incriminating evidence was presented last.

GPT-5.2 showed a weaker and more order-dependent version of this effect. Requiring an intermediate judgment increased final guilt ratings primarily when incriminating evidence was presented last. Thus, GPT-5.2 did not display the uniform measurement bias observed in GPT-4o, but instead showed an intermediate pattern between the GPT-4o measurement bias and the human evaluation bias.

Regarding both order effects and evaluation biases, we treat the observed patterns as descriptive effects and do not attempt to specify underlying mechanisms. For human behavior, a range of cognitive accounts have been proposed to explain both order effects and evaluation biases, and some theoretical frameworks explicitly link the two. A recent overview of these proposals is provided by Wojciechowski et al. (2025).

For GPT models, one possible interpretation is that changing the order of information introduces context effects or differing perspectives. The measurement effect or evaluation bias is more difficult to interpret at this preliminary stage without targeted experimental manipulations beyond the present design. One possibility is that the effect reflects self-conditioning or anchoring on the model’s earlier output, rather than an evaluative effect from the intermediate judgment as such. Distinguishing between these explanations would require additional control conditions—for example, an intermediate task unrelated to guilt—which were beyond the scope of the present work. In the current task, the intermediate question directly required an additional guilt assessment, making it unclear whether the observed effect reflects properties of the intermediate judgment itself or more general consequences of introducing an extra response step (a closely related possibility has already been examined in humans; see White et al., 2014).

Regarding GPT models, future studies could address this by modifying the intermediate stage. For example, asking a neutral question unrelated to guilt would test whether the bias arises simply from responding twice rather than from evaluating guilt at an intermediate step. If the effect persists, this would suggest that the structure of the task itself influences final judgments. If it disappears, this would indicate that requiring an intermediate guilt evaluation is critical for producing the effect.

While a consistent order effect was observed in all GPT models and prompt variants, this was not the case for evaluation biases. Although a related pattern was observed in both GPT-4o and GPT-5.2, its expression differed across model generations. In GPT-5.2, the effect was weaker and more dependent on evidence order, suggesting a partial attenuation relative to GPT-4o. This suggests that evaluation biases are not a fixed property of GPT models, but can vary across model generations.

Third, prompt engineering had limited and inconsistent effects. While some prompt variants changed the size or form of order and evaluation biases, none eliminated sensitivity to evidence order or reliably reduced evaluation biases.

In GPT-4o, prompt engineering led to only modest changes. One prompt variant reduced the size of the order effect, but a statistically significant order effect remained under all prompts. Importantly, none of the prompt variants altered the measurement bias: across all prompts, requiring an intermediate judgment continued to increase final guilt ratings, regardless of evidence order.

In GPT-5.2, prompt engineering had a stronger influence on how biases were expressed, but not in a consistently normative direction. Some prompts increased order effects, while others altered how the rating procedure interacted with evidence order. In particular, one prompt variant produced an order-dependent reversal, with the result resembling the evaluation bias observed in humans, whereas other variants increased the evaluation biases relative to the baseline condition in other ways.

Taken together, these findings indicate that prompt engineering can modify how biases manifest, but its effects are limited and unreliable. Simple changes to task instructions do not provide a robust safeguard against biased judgments in evaluative tasks, at least as far as the kind of legal decision-making we explored here is concerned. More broadly, these results should not be interpreted as demonstrating that prompt engineering cannot mitigate bias in general, but rather that the specific prompting strategies examined here did not reliably eliminate procedural sensitivities in this task.

Our investigation of prompt engineering was deliberately constrained. We focused on instruction-level changes that preserved the overall structure of the task and therefore did not include approaches such as chain-of-thought prompting. Such approaches introduce additional reasoning steps that overlap with the experimental manipulation of intermediate judgments and would require a different experimental design. Additionally, it is always possible that combinations of prompt variants might have a cumulative or interactive effect; the possible variants that one could usefully explore are unlimited. Our choice to focus on one prompt engineering manipulation at a time represents a choice of methodological simplicity.

### 4.1. Limitations and Future Directions

Several limitations should be acknowledged. First, the legal materials used in this study were simplified and cannot capture the full complexity of real-world legal decision-making. This simplification was necessary to maintain experimental control and comparability with prior human research, but it limits the direct applicability of the findings to real judicial contexts.

Second, reversing the order of incriminating and exonerating evidence necessarily also changes where this information appears within the model prompt. In other words, evidence that appears earlier in one condition appears later when the order is reversed. As a result, the manipulation combines changes in evidence order with changes in prompt position, as in the original experimental paradigm used by Wojciechowski et al. (2025). Future work could explore additional control conditions (e.g., padding or filler text) designed to better separate order effects from positional influences in language model prompts.

Third, although we examined two model versions (GPT-4o and GPT-5.2), the generalizability of the observed bias patterns remains an open question. Differences between these two models suggest that the strength and form of biases can change across model generations, but broader testing across additional models is needed to assess how widely these findings generalize. The primary analyses were conducted using a fixed temperature setting (the default value of 1) in order to isolate the effects of evidence order, rating condition, and prompt engineering. As a robustness check, we repeated the baseline analyses at a lower temperature (T=0.2), which yielded highly similar judgments across temperature settings. Nevertheless, because temperature can influence response variability, future work should examine more systematically how varying this parameter affects the expression of order effects and evaluation biases.

Finally, comparisons between humans and models were limited to the baseline condition. Prompt-engineered instructions were not administered to human participants, and differences between prompt-engineered model conditions and human judgments should therefore not be interpreted as direct performance comparisons. More generally, comparisons between human participants and large language models necessarily involve procedural asymmetries (e.g., the use of system prompts for models and the absence of attentional limits or fatigue). In the present study, we attempted to minimize such differences by closely matching the task structure and materials across agents, but the comparison should nevertheless be interpreted as assessing qualitative similarities in bias patterns rather than strict equivalence in decision processes. More broadly, establishing fully equivalent testing conditions between humans and large language models remains an open methodological question.

### 4.2. Normative and Ethical Implications

The present findings raise concerns about the use of GPT models in legal decision-making. Our results show that these models exhibit systematic biases, including order effects and rating-related distortions, and that such biases are not reliably eliminated by simple prompt-based interventions.

A key risk is not that GPT would formally replace human decision-makers, but that its outputs may be mistakenly treated as especially rational or objective. If relied upon without sufficient caution, GPT-based advice could influence judgments while masking its own limitations. At scale, the use of a shared system with consistent bias patterns also raises the risk of amplifying, rather than reducing, biased decision-making.

Importantly, responsibility for legal judgments necessarily remains with human users. However, meaningful responsibility requires awareness of the limits and biases of the tools being used. In light of the present findings, the use of GPT models in legal judgment should therefore be approached with caution, and not assumed to improve decision quality in the absence of robust, empirically validated safeguards.

## Figures and Tables

**Table 1:** Descriptive statistics for final guilt ratings from GPT-4o in the PE0 (baseline) condition, broken down by order and rating condition. *n* = number of observations; *M* = mean; SD = standard deviation.

Order	Rating Condition	*n*	*M*	*SD*
G–I	double	181	4.24	2.28
G–I	single	175	3.54	2.44
I–G	double	184	6.04	2.01
I–G	single	180	4.77	2.31

**Table 2:** Descriptive statistics for final guilt ratings from GPT-5.2 in the PE0 (baseline) condition, broken down by order and rating condition. *n* = number of observations; *M* = mean; SD = standard deviation.

Order	Rating Condition	*n*	*M*	*SD*
G–I	double	174	4.75	2.42
G–I	single	198	4.79	2.50
I–G	double	172	5.35	2.28
I–G	single	176	5.02	2.26

**Table 3:** Descriptive statistics for final guilt ratings from human participants, broken down by order and rating condition. *n* = number of observations; *M* = mean; SD = standard deviation.

Order	Rating Condition	*n*	*M*	*SD*
G–I	double	177	4.00	2.83
G–I	single	190	4.54	2.79
I–G	double	182	5.77	3.12
I–G	single	166	5.38	2.97

**Figure A1:**
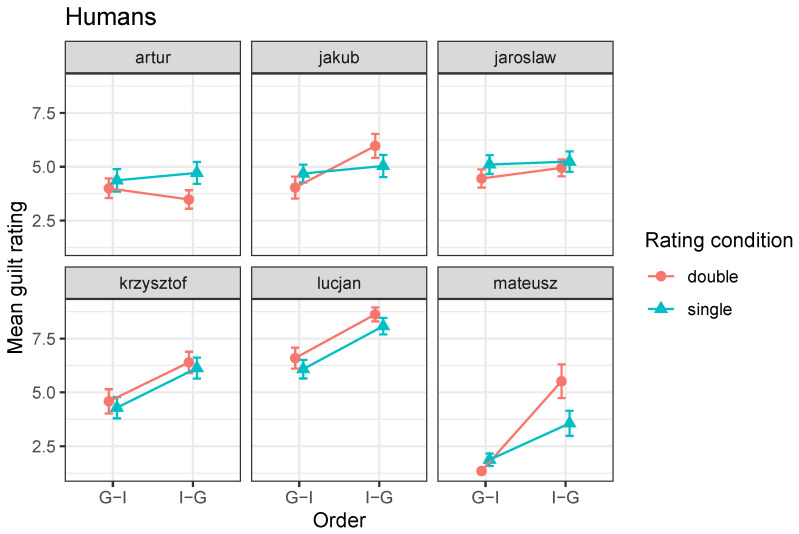
Mean final guilt ratings by case for human participants, shown as a function of evidence order (G–I vs. I–G) and rating condition (single vs. double). Error bars represent ±1 standard error of the mean.

**Figure A2:**
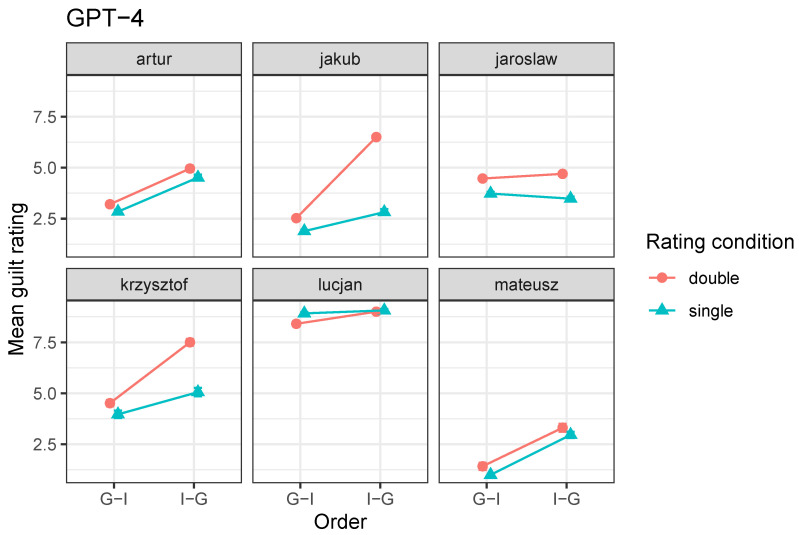
Mean final guilt ratings by case for GPT-4o in the baseline (PE 0) condition, shown as a function of evidence order (G–I vs. I–G) and rating condition (single vs. double). Error bars represent ±1 standard error of the mean.

**Figure A3:**
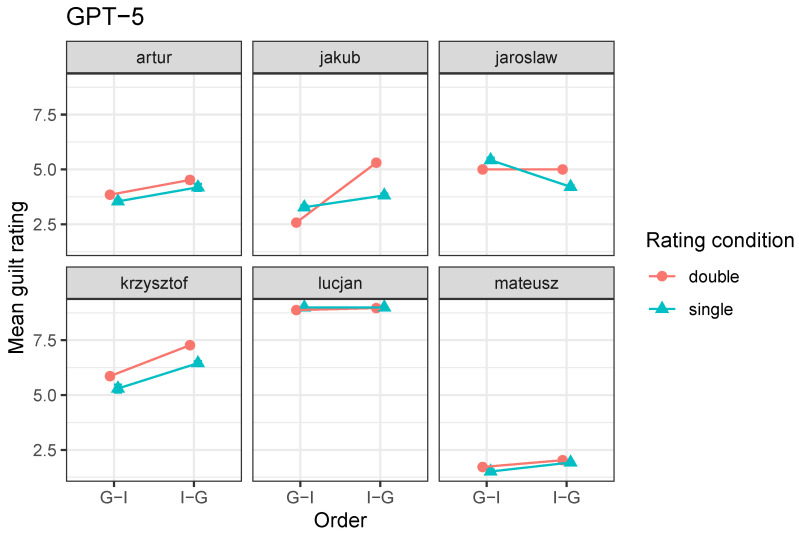
Mean final guilt ratings by case for GPT-5.2 in the baseline (PE 0) condition, shown as a function of evidence order (G–I vs. I–G) and rating condition (single vs. double). Error bars represent ±1 standard error of the mean.

## Data Availability

The data, analysis code, experimental materials, and scripts used to generate the results reported in this study are publicly available in the following GitHub repository: https://github.com/tfbessis/Legal-Biases-in-GPT (accessed on 14 April 2025).

## Appendix A. Case-Level Results

Figure A1, Figure A2 and Figure A3 present mean final guilt ratings by case for human participants, GPT-4o, and GPT-5.2, respectively. For each agent, results are shown as a function of evidence order (G–I vs. I–G) and rating condition (single vs. double). Error bars represent ±1 standard error of the mean.
